# Foot Plantar Pressure Measurement System Using Highly Sensitive Crack-Based Sensor

**DOI:** 10.3390/s19245504

**Published:** 2019-12-13

**Authors:** Jieun Park, Minho Kim, Insic Hong, Taewi Kim, Eunhan Lee, Eun-a Kim, Jae-Kwan Ryu, YongJin Jo, Jeehoon Koo, Seungyong Han, Je-sung Koh, Daeshik Kang

**Affiliations:** 1 Department of Mechanical Engineering, Ajou University, San 5, Woncheon-dong, Yeongtong-gu, Suwon 443-749, Korea; n9near@ajou.ac.kr (J.P.); hgh7706@ajou.ac.kr (M.K.); guyehl@ajou.ac.kr (E.L.); kya6758@ajou.ac.kr (E.-a.K.); sy84han@ajou.ac.kr (S.H.); 2 Unmanned/Robotics Team 1, LIG Nex1, 207, Mabuk-ro, Giheung-gu, Yongin 446-798, Korea; jaekwan.ryu@lignex1.com (J.-K.R.); yongjin.jo@lignex1.com (Y.J.); jeehoon.koo@lignex1.com (J.K.)

**Keywords:** foot plantar pressure, pressure measurement system, crack-based sensor, insole pressure sensor

## Abstract

Measuring the foot plantar pressure has the potential to be an important tool in many areas such as enhancing sports performance, diagnosing diseases, and rehabilitation. In general, the plantar pressure sensor should have robustness, durability, and high repeatability, as it should measure the pressure due to body weight. Here, we present a novel insole foot plantar pressure sensor using a highly sensitive crack-based strain sensor. The sensor is made of elastomer, stainless steel, a crack-based sensor, and a 3D-printed frame. Insoles are made of elastomer with Shore A 40, which is used as part of the sensor, to distribute the load to the sensor. The 3D-printed frame and stainless steel prevent breakage of the crack-based sensor and enable elastic behavior. The sensor response is highly repeatable and shows excellent durability even after 20,000 cycles. We show that the insole pressure sensor can be used as a real-time monitoring system using the pressure visualization program.

## 1. Introduction

Recently, measurement systems for foot plantar pressure are gaining attention in biomedical and sports-related research fields, such as ergonomic footwear design [1], sports performance analysis [2] and injury prevention, improvement in balance control [3], physical therapy, rehabilitation training systems [4,5,6], and disease diagnosis [7]. Monitoring foot plantar pressure distribution during daily activities provides a lot of useful biometric information related to human health condition. Analysis of this information helps us to develop personal-optimized footwear, enhance sports performance, monitor the rehabilitation state of a patient, and even detect diabetic foot ulceration early. To obtain the information effectively and accurately, a variety of plantar pressure measurement systems have been reported. In general, they can be classified into two types, platform systems [8,9,10,11] and in-shoe systems, which have advantages of long-term usage and mobility, respectively [12,13,14,15,16,17,18,19,20,21,22,23,24,25]. However, in-shoe systems are receiving more attention than platform systems these days due to their extensive utility. The systems maintain their functionality under repeated and sometimes harsh deformations from daily activities, while not causing any discomfort from wearing them. To endeavor to develop a reliable and comfortable foot plantar pressure monitoring system, many studies have been conducted. Previous measurement systems have many strengths in miniaturization [26], low-power consumption [27,28], and wireless setup [29,30], but neither durability nor sufficient sensitivity has been reported. Many high-sensitivity sensors have been studied, but they are not suitable to withstand the pressure from body weight and daily activities [31,32,33]. Here, we present a foot plantar pressure sensing system that is robust, highly sensitive, and easy to make. An insole made from silicone elastomer plays a role not only of a damper that improves robustness by distributing loads on the sensor but also as a part of the sensor. A rigid 3D-printed plastic and stainless steel sheets reinforced the robustness of our system, and high sensitivity is ensured through the crack-based sensor. We anticipate that our advanced pressure sensing system will provide versatile applications in many different areas.

## 2. Materials and Methods

### 2.1. Schematics of the Insole Pressure Sensor

**Figure 1:**
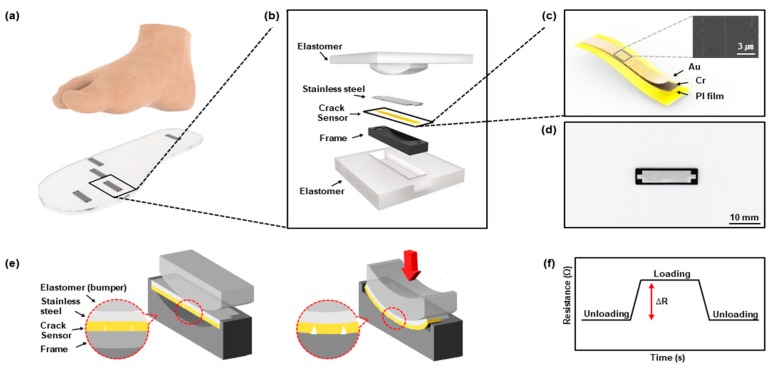
Schematic and principle of foot plantar pressure using a highly sensitive crack-based sensor: (**a**) Schematic illustration of the crack-based, sensor-based insole plantar pressure sensor; (**b**) separation diagram of the sensor; (**c**) schematic illustration of the crack-based sensor. The inset image presents an SEM image of cracks on the sensor; (**d**) image of stainless steel with the crack-based sensor attached in the frame; (**e**) schematic illustration of the sensing mechanism; (**f**) resistance changes in responses to loading and unloading.

The foot plantar pressure measurement system uses an insole pressure sensor (Figure 1a). The insole is made of an elastomer with a hardness of Shore A 40 commonly used for insoles [34,35]. The pressure sensor consists of stainless steel, a crack-based sensor, a frame, and the elastomer (Figure 1b). The crack-based sensor, inspired by a spider’s sensory apparatus, is an ultra-high sensitivity strain sensor that uses changes in resistance when the nanocracks in the metal layer on the polymer substrate are disconnected and reconnected by external forces [36]. The crack-based sensor in the pressure sensor consists of three layers as shown in Figure 1c: a gold top layer as an electrical conductor, a chrome layer as a crack-generating layer, and a 7.5 µm thick polyimide (PI) film as a substrate. This three-layered sensor is attached to the stainless steel to prevent damage by external force and to elicit elastic behavior when external force is applied and removed. The 3D-printed frame is designed to make the crack-based sensor attached to the stainless steel deform only by the normal-direction force. The crack-based sensor attached to the stainless steel can be inserted and fixed in the frame (Figure 1d), and the frame has a chamber with a specific radius of curvature. The sensor’s upper elastomer has a bumper which has the same radius of curvature with the frame. The upper elastomer and the bumper distribute the load on the crack-based sensor attached to the stainless steel while eliminating any inconvenience that may arise when pressing the 3D-printed frame and stainless-steel parts, which are rigid. If the crack-based sensor attached to the stainless steel is bent to pressure, the nanocracks of the crack-based sensor disconnect and the resistance increases (Figure 1e), so the pressure can be detected as a change in resistance (Figure 1f).

### 2.2. Fabrication

We used a 7.5 µm thick PI film (3022-5 Kapton thin film, Chemplex, Palm City, FL, USA) as a substrate of the crack-based sensor. According to a previous study, the depth and density of cracks are important factors that determine the sensor’s sensitivity, and the thickness of the chromium is closely related to the depth of cracks [37,38]. Sensors have high sensitivity when the chromium layer is 60 nm thick and the gold layer is 20 nm thick, due to deep cracks and the low crack density effect (performance degradation due to a small number of cracks). Therefore, we sequentially deposited 60 nm thick chromium and 20 nm thick gold by using the thermal evaporating system (Thermal Evaporation System, DD High Tech. Co., Gimpo-si, Gyeonggi-do, Korea). The metal-deposited PI film, then, was stretched to generate cracks by 2% at a rate of 40 mm/min by using a material testing machine (3342 UTM, Instron Co., Norwood, MA, USA). This process was repeated for about 500 cycles until the changes in the resistance of the sensor converged. A sheet of 304 stainless steel was cut by Compact Laser Micromachining system (A series, Oxford lasers Inc., Shirley, MA, USA) as shown in Figure S1. The surface of the steel was covered with a layer that contained detrimental materials such as processing lubricant, mill scale, and oxidation products, which may have had deleterious effects on adhesive bonds. Hence, the surface layer was removed through acid pickling, and then the crack-based sensor was attached [39,40,41]. The crack-based sensor and acid-cleaned 304 stainless steel sheets were bonded together by using a strain gauge adhesive (Type CN, Tokyo Sokki Kenkyujo Co., Tokyo, Japan). When attaching the crack-based sensor to the stainless steel, only the PI film of the sensor was attached to the stainless steel, as shown in Figure S2, to prevent a short-out due to the connection between the gold layer for electrical conductors and stainless steel. The frame part was made by a 3D printer system (ProJet MJP 2500, 3D Systems, Rock Hill, SC, USA). The specific dimensions of the frame are provided in Figures S1 and S3. We used a UV-curable plastic (VisiJet M2 RBK, 3D Systems, Rock Hill, SC, USA) and wax support material (VisiJet M2 SUP, 3D Systems, Rock Hill, SC, USA) for 3D printer material. When the printing process was completed, the frame was washed with hot mineral oil to remove any support wax remaining on the outside and inside of the frame. The elastomer part was fabricated with a silicon elastomer (Mold Master hard, MOLKANG, Paju-si, Gyeonggi-do, Korea). A silicon base and hardening agent were mixed for 3 min and then cured for 2 h in a constant temperature chamber at 70 ℃. The specific dimensions of he telastomer part are provided in Figure S3. To make the insole pressure sensor, we placed the pressure sensor and injected additional elastomer under the heel and the 1st, 3rd and 5th metatarsals, and the great toe, because the different locations of the sensor may help differentiate the various aspects of the gait cycle [42,43,44].

### 2.3. Pressure Sensor Test Method

To verify the performance of the pressure sensor, a loading test was performed by using a material testing machine (3342 UTM, Instron Co., Norwood, MA, USA) at a sampling rate of 500 Hz. The load test was performed before the pressure sensor became the insole. After attaching and pressing a plate with a larger area than the sensor, the pressure was calculated by dividing the force of the material testing machine by the sensor area (A = 7.0 × 10^−4^ m^2^) (Figure S4). To measure the pressure, we used a conductive epoxy to attach Teflon-coated wires on the gold layer as electrical conductors of the crack-based sensor that detects resistance variation according to the pressure (Figure S5). The resistance variation data were gathered by the data acquisition system (DAQ) (SIRIUS, Dewesoft doo, Trbovlje, Slovenia) at a sampling rate of 500 Hz. The data from the DAQ and the material testing machine were combined to obtain resistance data for pressure in the sensor. In this regard, obtaining data from the material testing machine and the DAQ with the same sample rate makes the data easier to combine. In order to make the sensor with optimal performance, experiments were conducted using the thickness of the stainless steel (*d_m_*) and the radius of curvature of the frame (*r*) as variables (Figure S3).

## 3. Results

### 3.1. Parameter Study to Design Pressure Sensor

The crack-based sensor shows resistance variation according to the strain applied to the metal layer. The pressure sensor is only affected by the strain due to bending since the crack-based sensor is attached to stainless steel and fitted into the frame. Therefore, understanding the strain according to the degree of bending is important for making the sensor with optimal performance. We designed the sensor suitable for measuring foot plantar pressure through the parameter study of *d_m_* and *r*. When the crack-based pressure sensor is bent, the bending strain of the metal layer is expressed as in the Equation (1) [45,46,47] with an assumption that the thickness of the metal layer (~80 nm) can be neglected.

**Figure 2:**
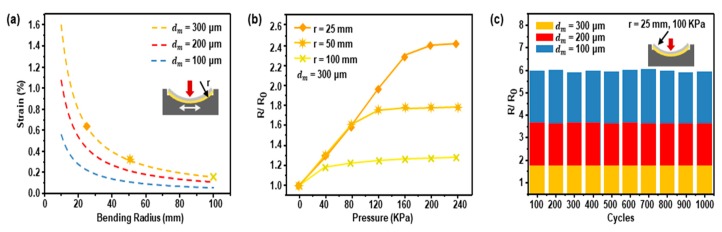
Parameter study to design pressure sensor: (**a**) theoretical strain of metal layer of the crack-based sensor according to thickness of the stainless steel (*d_m_*) and the radius of curvature of the frame (*r*); (**b**) resistance variation with radius of curvature when *d_m_* is 300 µm; (**c**) performance difference by *d_m_* with *r* = 25 mm and pressed at 100 kPa.

ε = ((*d_m_* + *d_f_*)/2*r*) × ((1 + 2*η* + *χη*^2^))/(1 + *η*)(1 + *χη*), (1)

where *η* = *d_m_*/*d_f_*, Young’s modulus of stainless steel is *Y_m_*, PI film is *Y_f_*, and *χ* = *Y_m_*/*Y_f_*. Properties are shown in Table S1. Figure 2a shows the theoretical strain within the metal layer on bending PI according to the *r* and *d_m_*. Decreasing *d_m_* reduces the strain applied to the metal layer, which then results in the deterioration of the sensitivity of the sensor. The sensitivity, on the other hand, is improved when *r* decreases because the strain applied to the metal layer is increased. Theoretically, a small *r* makes the sensitivity better because the strain applied to the metal layer on the crack-based sensor becomes larger. The minimum *r*, which can be determined by considering the overall thickness of the sensor and the dimensions inside and outside the frame, is 13.85 mm. However, if *r* is 13.85 mm, the thinnest part between the bottom of the radius of curvature and the frame (*a*) (see Figure S3) can cause the frame to break when pressure is applied. The minimum thickness to prevent breakage due to *a* is 1 mm where *r* is 25 mm. Figure 2b shows a graph of the resistance variation with pressure when it is applied up to 250 kPa. Fixing *d_m_* to 300 µm, the sensor’s resistance variation increases when the *r* is decreased. When *r* is 25, 50, and 100 mm, it can detect up to 160, 80, and 40 kPa, respectively, because *r* makes the metal layer on the crack-based sensor have a strain of 0.6%, 0.3%, and 0.1%, respectively, and limits further bending of the crack-based sensors attached to stainless steel.

The normalized resistance variation for each *d_m_* with the optimized value (*r* = 25 mm) is shown in Figure 2c. When 100 kPa is applied to the sensor, a *d_m_* of 100 µm has the largest resistance variation because the stainless steel has a smaller radius of curvature than the *r* due to its low stiffness (Figure S6). This results in stress concentrations where cracks in the crack-based sensor are easily deepened and degrade sensor performance. In addition, the thin *d_m_* causes plastic deformation to induce prestrain on the metal layer of crack-based sensor even when the pressure is removed. Because of these problems, the use of a thin *d_m_* is not suitable for repeatability and durability of the sensor. Thus, the optimized *d_m_* value can be decided as 300 µm.

### 3.2. Pressure Sensor Performance

**Figure 3:**
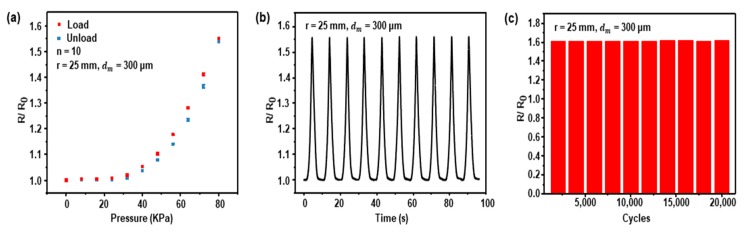
The pressure sensor performance test with *r* of 25 mm, *d_m_* of 300 µm: (**a**) hysteresis curve of the sensor when the sensor receives loading/unloading 0 to 80 kPa pressure; (**b**) the variations of the performance of the sensor; (**c**) the durability of the sensor with 20,000 cycles.

Maintaining performance during long-term iterative and sometimes harsh deformation of daily activity is one of the most essential requirements for foot plantar pressure sensors. To check the repeatability and durability, we performed cycling experiments with optimized sensors (*r* = 25 mm, *d_m_* = 300 µm) that were found through parametric study. Figure 3a shows the loading and unloading tests of the pressure sensor and the normalized resistance versus pressure curve averaged over ten of the same samples of the sensor. Red dots represent loading the sensor to the final pressure of 80 kPa, and blue dots represent unloading. The hysteresis of loading/unloading was small. Figure 3b shows a representative set of 10 cycles that have consistent resistance variation. We repeatedly pressed the sensor with 0 to 80 kPa and 40 mm/min velocity. In the durability test, we tested more than 20,000 cycles. The resistance variation gradually increased initially but converged. Normalized resistance changes showed stability at 5000 cycles and additional cycles (Figure 3c).

### 3.3. Foot Plantar Pressure Measurement System

**Figure 4:**
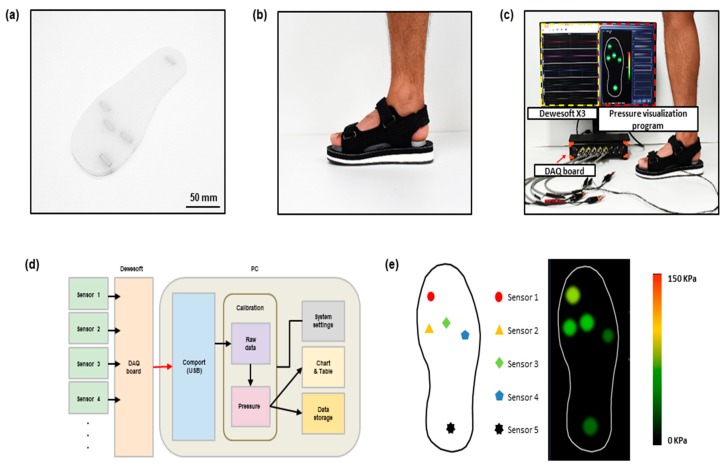
Schematic and image of foot plantar pressure measurement system: (**a**) conceptual photograph of the crack-based, sensor-based insole plantar pressure sensor; (**b**) conceptual photograph of a shoe with the insole inserted in it; (**c**) measurement configuration of the crack-based, sensor-based plantar pressure sensor; (**d**) overview of the system architecture; (**e**) sensor position and pressure visualization program.

Figure 4a,b show the conceptual photograph of the insole pressure sensor and an image of the sensor placed in a shoe. The entire system of the foot plantar pressure measurement in operation is shown in Figure 4c. The resistance data from the sensor are sent to the computer via the DAQ board. Dewesoft X3 (programs associated with the DAQ board) shows the resistance data. For calibration, we replaced the resistance data for pressure (see Figure 3a) with the pressure data for resistance, and then the power law was used to make a theoretical equation. The power law calibrates the resistance by the Dewesoft X3 to be displayed in pressure. The calibrated pressure data are shown in the pressure visualization program (Figure 4d). Basically, a sensing area is under the heel, the 1st, 3rd, and 5th metatarsals, and the great toe, but the location of the sensing area may change depending on the participant, and also the measuring area may be different. Pressure can be monitored by colorimetric change in a pressure visualization program (Figure 4e).

### 3.4. Measured Pressure during Gait Cycle

**Figure 5:**
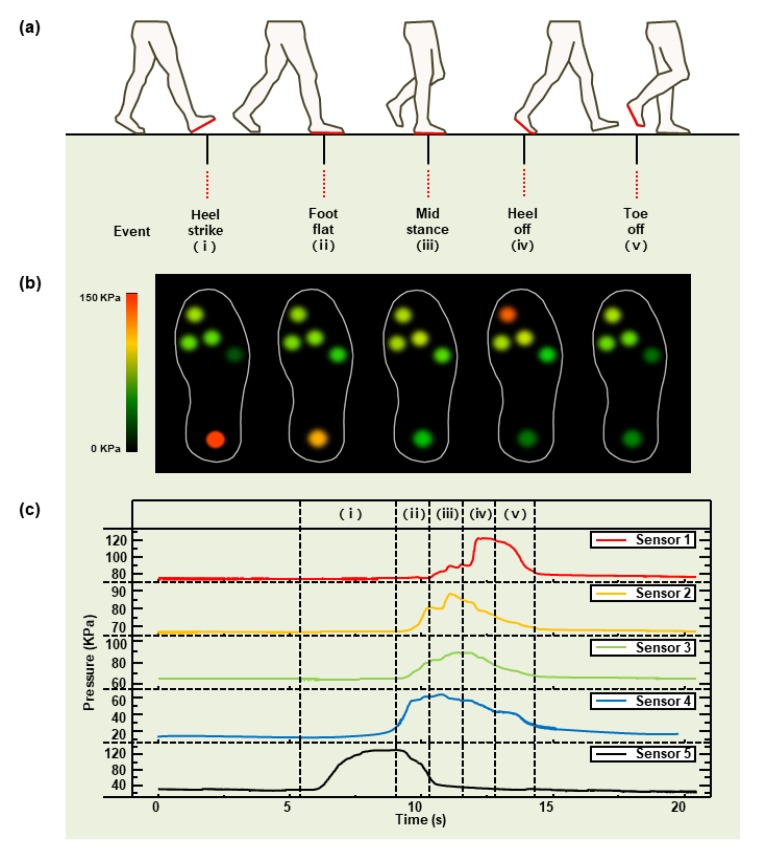
Pressure measurement during stance phases of a gait cycle: (**a**) schematic of insole plantar pressure measurement during the walking phases and events of a half gait cycle; (**b**) pressure visualization program during a half gait cycle (right foot); (**c**) graph for each walking phase.

Figure 5a shows stance phases of a gait cycle, which are heel-strike, foot-flat, mid-stance, heel-off, and toe-off. In accordance with these phases, the measurement was taken from the right foot of a male participant weighing 72 kg. The pressure changes of the five areas are visualized and shown in real time. Through the visualization program, the distribution of pressure on the foot plantar during the gait cycle is shown as a colorimetric change (Figure 5b). The pressure-to-time graph is shown in Figure 5c. The biggest pressure during the gait cycle occurred when the body weight was supported by the heel and the great toe. The pressure at that time was around 120 kPa. Sensor 5 under the heel and Sensor 1 under the great toe measured the maximum pressure at the heel-strike stage and heel-off stage, respectively, and the measured pressure was about 120 kPa. The maximum pressures of Sensors 2, 3, and 4 under the metatarsals were shown in the mid-stance stage, and their values were 90, 90, and 60 kPa, respectively.

## 4. Discussion

In summary, a foot plantar measurement system that uses a highly sensitive crack-based sensor for real-time monitoring of plantar pressure with visualization has been presented. This insole pressure sensor can measure up to 160 kPa in five or more parts of the plantar area and can be customized to the participant. It has a highly repeatable response and boasts high durability, even after 20,000 cycles. These results indicate that our sensor has outstanding advantages in performance compared to other insole foot plantar pressure sensors and monitoring systems previously introduced. Therefore, our foot plantar pressure measurement system, with its advantages of suitability, durability, and real-time monitoring system, has a great potential to be utilized in various fields requiring foot plantar pressure, such as balance control, physical therapy, rehabilitation systems, diagnosis of disease, and sports-related research fields. In the future, the use of an insole type pressure sensor in diverse fields requires the development of a portable DAQ board and wireless data transmission, reliability improvement in the mass production process, as well as pressure visualization programs for use on smartphones.

## Supplementary Materials

The following are available online at https://www.mdpi.com/1424-8220/19/24/5504/s1. Figure S1: Specific dimensions of the frame and the stainless steel; Figure S2: Schematic illustration of the crack-based sensor and stainless steel attachment; Figure S3: Cross-sectional image of the sensor and specific dimensions of the elastomer and inside the frame; Figure S4: Schematic illustration of the experimental setup; Figure S5: Schematic illustration of a wire-connected crack-based sensor; Figure S6: Curvature radius difference due to stiffness difference; Table S1: Material properties of stainless steel, crack-based sensor (PI film).
