# Supplementary material for: Foot Plantar Pressure Measurement System Using Highly Sensitive Crack-Based Sensor
Source: Sensors (Basel). 2019 Dec 13;19(24):5504. doi: 10.3390/s19245504 (PMC6960515; doi:10.3390/s19245504)
Supplement: Supplementary file 1 [file sensors-19-05504-s001.pdf]

sensors

Supplementary Materials

Foot Plantar Pressure Measurement System Using Highly Sensitive Crack-Based Sensor

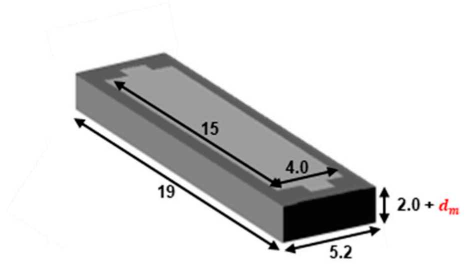

Figure S1. Specific dimension of the frame exterior and the stainless steel.

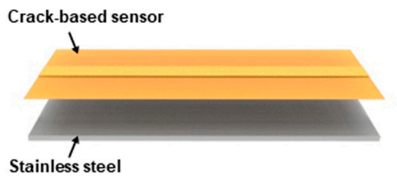

Figure S2. Schematic illustration of the crack-based sensor and stainless steel attachment.

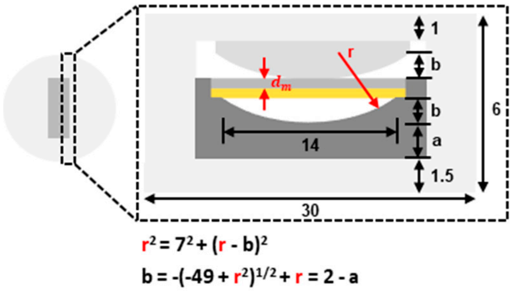

Figure S3. Cross-sectional image of the sensor and specific dimensions of the elastomer and inside the frame.

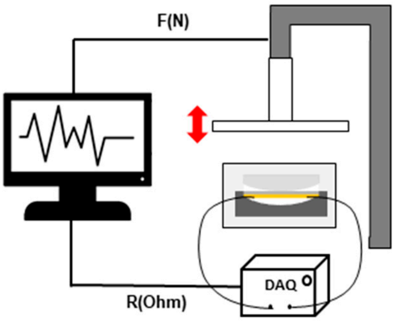

Figure S4. Schematic illustration of the experimental setup.

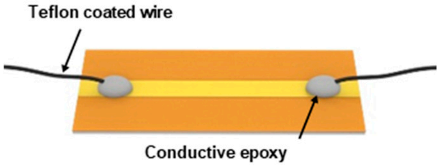

Figure S5. Schematic illustration of wire-connected crack-based sensor.

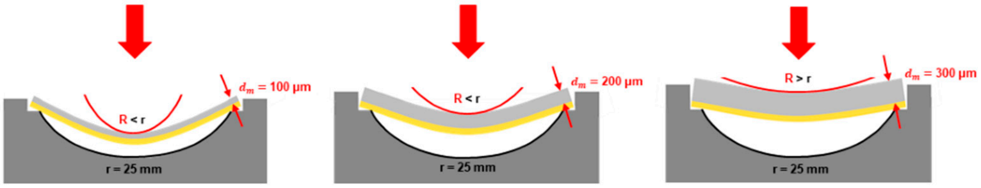

Figure S6. Curvature radius difference due to stiffness difference.

Table S1. Material properties of stainless steel, crack-based sensor (PI film).

	**Young's modulus**	**Thickness**
Stainless steel 304	200 GPa (Y_m)	d_m
Crack-based sensor (PI film)	2.5 GPa (Y_f)	8 μm (d_f)
